# Fully transparent flexible tin-doped zinc oxide thin film transistors fabricated on plastic substrate

**DOI:** 10.1038/srep38984

**Published:** 2016-12-12

**Authors:** Dedong Han, Yi Zhang, Yingying Cong, Wen Yu, Xing Zhang, Yi Wang

**Affiliations:** 1 Institute of Microelectronics, Peking University, Beijing 100871, China; 2 Shenzhen Graduate School, Peking University, Shenzhen 518055, China

## Abstract

In this work, we have successfully fabricated bottom gate fully transparent tin-doped zinc oxide thin film transistors (TZO TFTs) fabricated on flexible plastic substrate at low temperature by RF magnetron sputtering. The effect of O_2_/Ar gas flow ratio during channel deposition on the electrical properties of TZO TFTs was investigated, and we found that the O_2_/Ar gas flow ratio have a great influence on the electrical properties. TZO TFTs on flexible substrate has very nice electrical characteristics with a low off-state current (I_off_) of 3 pA, a high on/off current ratio of 2 × 10^7^, a high saturation mobility (μ_sat_) of 66.7 cm^2^/V•s, a steep subthreshold slope (SS) of 333 mV/decade and a threshold voltage (V_th_) of 1.2 V. Root-Mean-Square (RMS) roughness of TZO thin film is about 0.52 nm. The transmittance of TZO thin film is about 98%. These results highlight that the excellent device performance can be realized in TZO film and TZO TFT can be a promising candidate for flexible displays.

The next generation of flexible displays, based on organic light-emitting diodes (OLEDs), is expected to bring excellent view ability as well as a huge spectrum of colors. Recent years, more and more studies on oxide thin film transistors (TFTs) have been reported for their widely use in driving active matrix OLEDs. Zinc oxide (ZnO) based thin films are widely used to fabricate TFTs due to their good optical and electrical properties, and good uniformity and low process temperature^1^,^2^,^3^,^4^,^5^,^6^,^7^,^8^,^9^,^10^,^11^,^12^,^13^,^14^,^15^,^16^,^17^,^18^.

Among various ZnO-based TFTs, one of the most promising materials is indium gallium zinc oxide (IGZO), which shows high electron mobility. However, IGZO thin film requires high fabricating and annealing temperature. Besides, indium element is a rare element, and the storage of In is very limited on earth. The price of indium is very high and keeping increasing. It is predicated that there will probably be a shortage of In supply in the future. Moreover, In is poisonous and harmful to human health. So novel materials for channel should be studied. The indium-free oxide-based channel materials such as zinc tin oxide (ZTO) fabricated on glass have been extensively studied^3^,^4^,^5^. In this letter, we choose tin-doped zinc oxide (TZO) as our experiment material. Sn is non-toxic and abundant on earth. SnO_2_ film is reported to possess excellent electrical characteristics. In the paper, we developed TZO TFTs on flexible substrate and studied their electrical characteristics, which were different from that on glass substrate. We turned to research the electrical performance of TZO TFTs by adjusting oxygen partial pressure (OPP) during the fabrication of the TZO film as active layer. The experiment results showed better properties of TZO TFTs than all the papers reported before^3^,^4^,^5^,^19^,^20^,^21^,^22^,^23^. The device had a low off-state current value (I_off_) of 3 pA, a high on/off current ratio of 2 × 10^7^, a high saturation mobility (μ_sat_) of 66.7 cm^2^/V•s, a subthreshold swing (SS) of 333 mV/decade, and a threshold voltage V_th_ of 1.2 V. These pleasing results make TZO a promising material for transparent flat panel display.

## Results and Discussion

**Figure 1:**
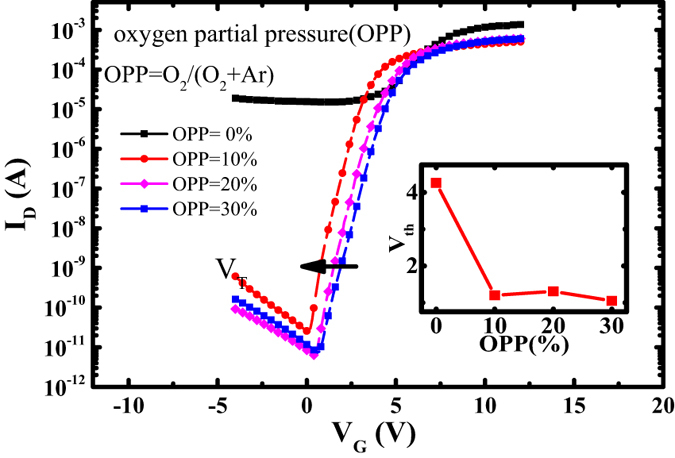
Transfer curves of the TZO TFT with various OPPs. The inset is threshold voltage of TZO TFTs.

Figure 1 illustrates the transfer characteristics of the bottom-gate TZO TFTs with various O_2_/Ar gas pressure ratios. We analyzed the electrical characteristics of the four samples by a semiconductor parametric analyzer (Agilent 4156 C) at room temperature. Figure 1 indicates the transfer characteristics of the samples at V_D_ = 5 V (W/L = 100 μm/50 μm). We could see that the electrical properties of the TZO TFTs are vastly improved after adding O_2_ during the growth of the TZO thin films but do not show large difference when the OPP increases. Table 1 shows the extracted electrical parameters of TZO TFTs. The threshold voltage (V_th_) is achieved by extrapolating the linear fitting to I_D_^1/2^-V_G_ plot under the condition V_D_ > V_G_ − V_th_, and we can calculate the saturation mobility (μ_sat_) through the slope of fitted line and equation (1). The subthreshold swing can be calculated by equation (2).


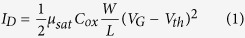



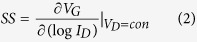


The mobility was extracted using transconductance method (G_m_ method):


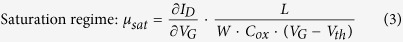


**Figure 2:**
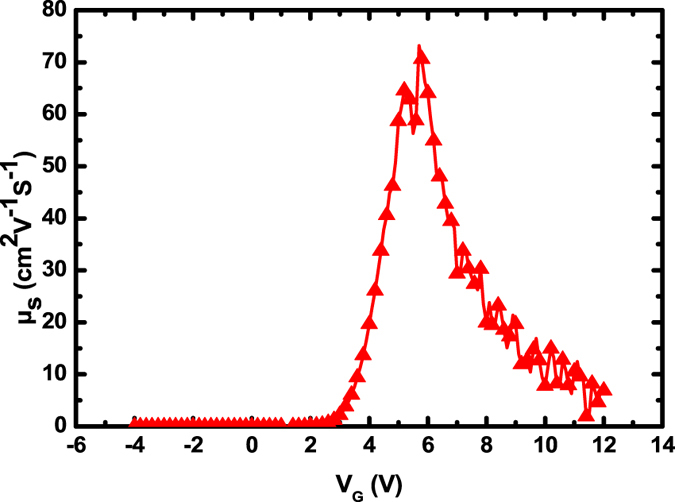
The saturation mobility (μ_s_) versus V_G_ curve of the TZO TFT.

Here, The capacitance per unit area C_OX_ is 2.6 × 10^−8^ F/cm^2^, which is extracted from C-V curve measured by Keithley590 C-V analyzer. V_D_ is 5 V. The saturation mobility (μ_s_) versus V_G_ curve is extracted from saturation regime using transconductance (Gm) method, which is shown in Fig. 2.

We can see from Fig. 1 that the I_on_ and I_off_ decrease with the increasing of OPP from 0% to 10%, but not obviously when the OPP keeps on increasing. This result stem from the variation of OPP which lead to the change of oxygen vacancy (V_o_) including lots of electrons, which weaken the gate control and cause the poor transfer curve. With OPP increasing, the devices have better performance as V_o_ decreases to reinforce gate control. However, excessive oxygen contents will also deteriorate the performance owing to its large resistance from move V_o_. While the OPP keeps on increasing, oxygen contents reach saturation, V_o_ do not decrease. So the TFT characteristic does not change so much^24^.

**Figure 3:**
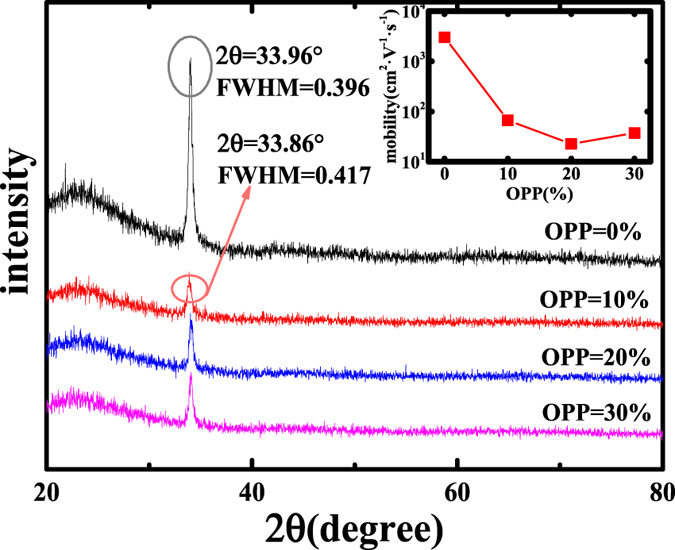
X-ray diffraction pattern of the TZO film sputter-deposited on glass substrate. The inset is saturation mobility of TZO TFTs with various OPPs.

**Figure 4:**
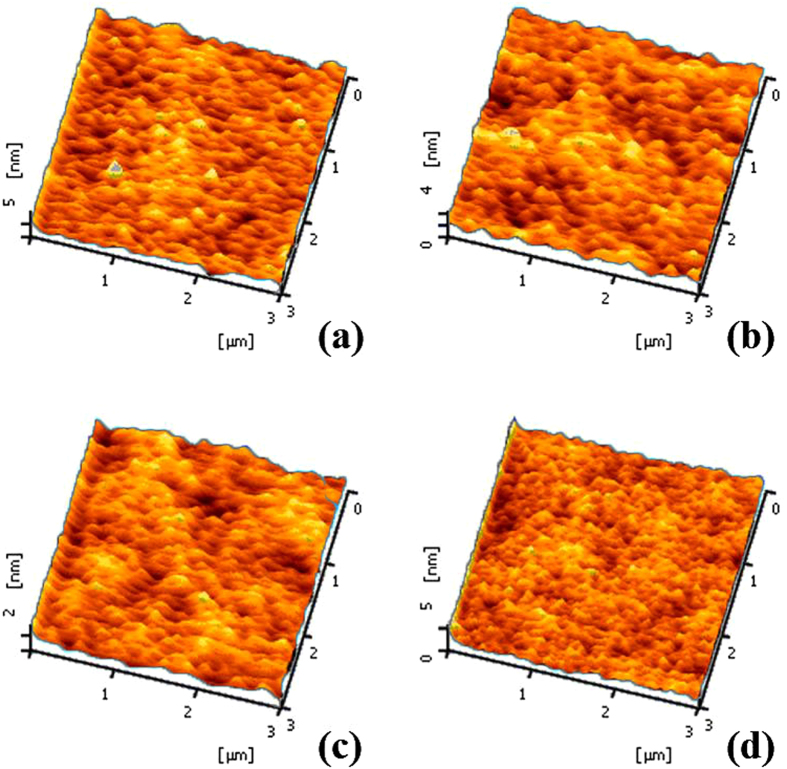
The AFM surface morphology of the TZO channel film with OPP of 0% (**a**), 10% (**b**), 20% (**c**) and 30% (**d**).

The XRD results of TZO thin film are shown in Fig. 3. The thin film was deposited by RF magnetron sputtering at RT on glass substrate. Four curves indicated different sputtering condition. There was only one peak at 2θ ≈ 34°, which mean the TZO was C-axis aligned crystalline (CAAC) oxide semiconductor^25^. The FWHM was measured as 0.417° (O_2_/Ar = 10/90) and 0.396° (O_2_/Ar = 0/100) respectively, which indicated the grain sizes of the film. Using the Scherer formula (D = (KΛ)/(Bcosθ)), we can get the average grain size: 20.7 nm (O_2_/Ar = 0/100) and 19.6 nm (O_2_/Ar = 10/90) respectively. This result was also confirmed by AFM surface morphology of the TZO surface (shown in Fig. 4), the extracted Root-Mean-Square (RMS) roughness of TZO thin films are shown in Table 2. We can see the TZO film (O_2_: Ar is 10:90) is compacter and more smoothing, the TZO thin film’s Root-Mean-Square (RMS) roughness is about 0.52 nm.

The high mobility TZO TFTs owe to optimizing process, Tin dopant concentrations, and O_2_/Ar gas pressure ratio. The role of oxygen during the deposition process is a key condition. Excessive oxygen may make I_on_ decrease, and Poor oxygen may make I_off_ increase. The process of SiO_2_ and TZO thin films continual growth is adopted to decrease interface-state density, and enhance mobility of TZO TFTs. The high thin film quality is also a way to improve the device characteristics performances.

**Figure 5:**
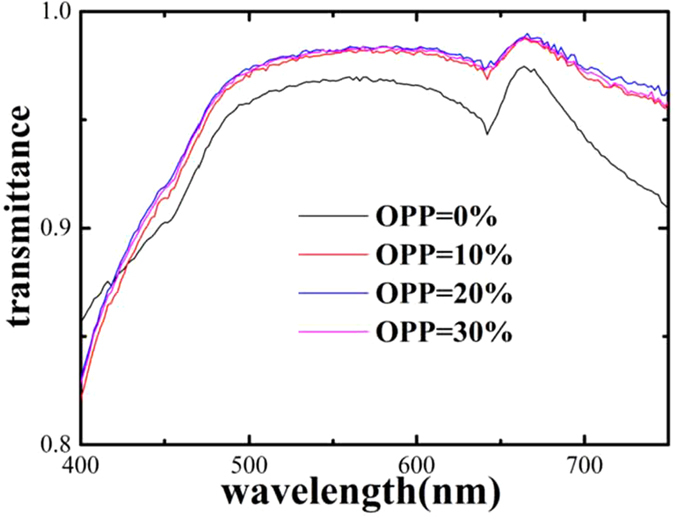
The transmittance of TZO TFTs with different OPPs.

It is shown in Fig. 5 that the transmittance of 10%-TZO films is about 98%, and TFT fabricated shows good transparency, attesting the devices are applicable for the transparent flexible display.

In conclusion, High-performance transparent bottom gate type Tin-doped ZnO TFTs had successfully fabricated by RF Magnetron Sputtering on flexible substrate at low temperature. We had investigated the effect of O_2_ gas partial pressure during active channel layer deposition on the electrical properties of the device. We found growing OPP for TZO films as the channel layer did not influence the TFT characteristic much. The TZO TFTs as-fabricated exhibited excellent electrical properties, with a high I_on_/I_off_ of 2 × 10^7^, a steep SS of 0.333 V/dec, a high μ_s_ of 66.7 cm^2^/V·s and a low V_th_ of 1.20 V, as the OPP was 10%. These results highlight TFTs on flexible substrates using TZO films as channel layer material can realize excellent device performance and TZO TFT can be a promising candidate for transparent flexible displays.

## Methods section

**Figure 6:**
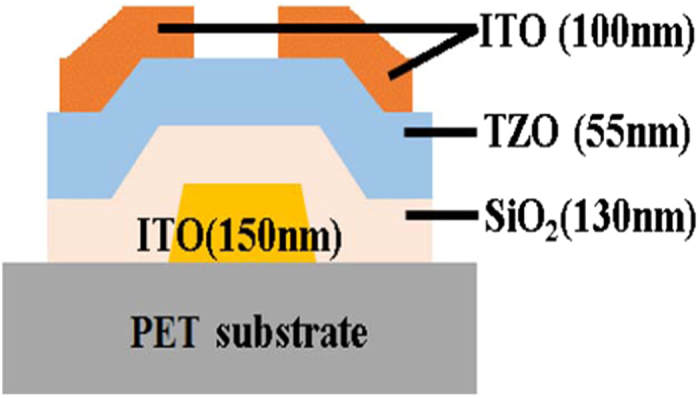
Cross-section view structure schematics of TZO TFTs.

In this experiment, bottom-gate type TZO TFT was fabricated by photolithography and lift-off technique. All the devices were fabricated on two-inch plastic substrates. The device structure is shown in Fig. 6. We used indium tin oxide (ITO) as gate electrode, TZO as the active layer, and ITO as S/D electrode, which were all deposited by RF sputtering. The gate insulator (SiO_2_) was deposited by plasma-enhanced chemical vapor deposition (PECVD). All the process temperatures are below 100 °C. Before each deposition step, the growth chamber was pre-pumped to the base vacuum pressure of 5 × 10^−4^ Pa. During each deposition step, the working pressure was kept at 1.0 Pa.

**Figure 7:**
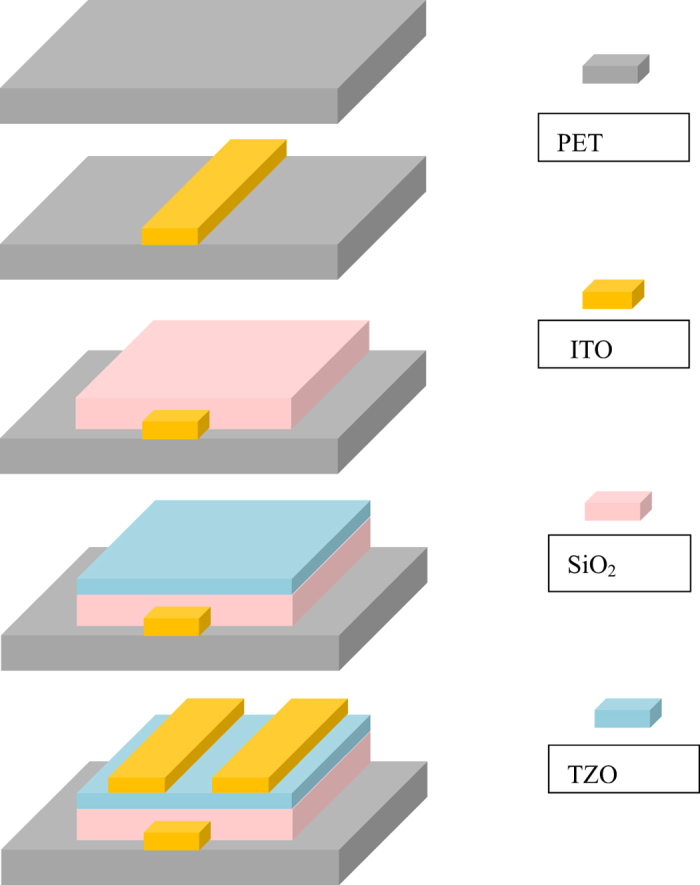
Schematic illustration of the TZO TFT fabrication scheme.

The fabrication procedures were as shown in Fig. 7. First, a gate electrode was patterned and a 150-nm-thick ITO film was deposited by RF sputtering on the plastic substrate, in pure Ar atmosphere at room temperature as a gate electrode. Next, a stack of 130-nm-thick gate insulator (SiO_2_) was fabricated on the gate electrode by PECVD at 80 °C. Third, a stack of 55-nm-thick channel layer (TZO) was sputtered at room temperature under a mixed atmosphere of Ar and O_2_. The RF power was kept at 70 W. The sputtering target of TZO thin film was an φ80 mm × 6 mm ceramic target with a mass ratio of ZnO:SnO_2_ = 97:3. Fourth, after patterning the source and drain electrode, a film of 100-nm-thick ITO was deposited using RF sputtering at room temperature and lifted to form the source and drain electrodes.

We investigate the effects of O_2_/Ar gas pressure ratio during channel deposition on the electrical characteristics of the TZO TFTs. There are four different samples, and their channel layers were deposited under the same total working pressure of 1.0 Pa but different O_2_/Ar gas pressure ratios (0:100, 10:90, 20:80, 30:70). After fabrication, the TFTs were electrically characterized by a semiconductor parameter analyzer (Agilent4156C). Crystallographic structure, and surface morphology of the TZO thin films were evaluated by x-ray diffraction (XRD) and atomic force microscopy (AFM). The transmittance of TZO with different OPPs were tested. Capacitance-voltage (C-V) characteristic was measured by Keithley590 C-V analyzer.

## Additional Information

**How to cite this article**: Han, D. *et al*. Fully transparent flexible tin-doped zinc oxide thin film transistors fabricated on plastic substrate. *Sci. Rep.* **6**, 38984; doi: 10.1038/srep38984 (2016).

**Publisher's note:** Springer Nature remains neutral with regard to jurisdictional claims in published maps and institutional affiliations.

## Figures and Tables

**Table 1:** Extracted parameters of TZO TFTs with various OPP.

Oxygen flow ratio	μ_s_ (cm^2^/V·s)	V_th_ (V)	SS (V/dec.)	On/off ratio
0:100	—	4.26	0.256	1 × 10^2^
10:90	66.7	1.20	0.333	2 × 10^7^
20:80	22.7	1.31	0.308	6 × 10^7^
30:70	37.4	1.05	0.345	6 × 10^7^

**Table 2:** Root-Mean-Square (RMS) roughness of TZO thin films with various OPP.

Oxygen flow ratio	0:100	10:90	20:80	30:70
RMS (nm)	1.1	0.52	0.45	0.56
